# Supplementary material for: Structural delineation and computational design of SARS-CoV-2-neutralizing antibodies against Omicron subvariants
Source: Nat Commun. 2023 Jul 14;14:4198. doi: 10.1038/s41467-023-39890-8 (PMC10349087; doi:10.1038/s41467-023-39890-8)
Supplement: Supplementary file 1 — Supplementary information [file 41467_2023_39890_MOESM1_ESM.pdf]

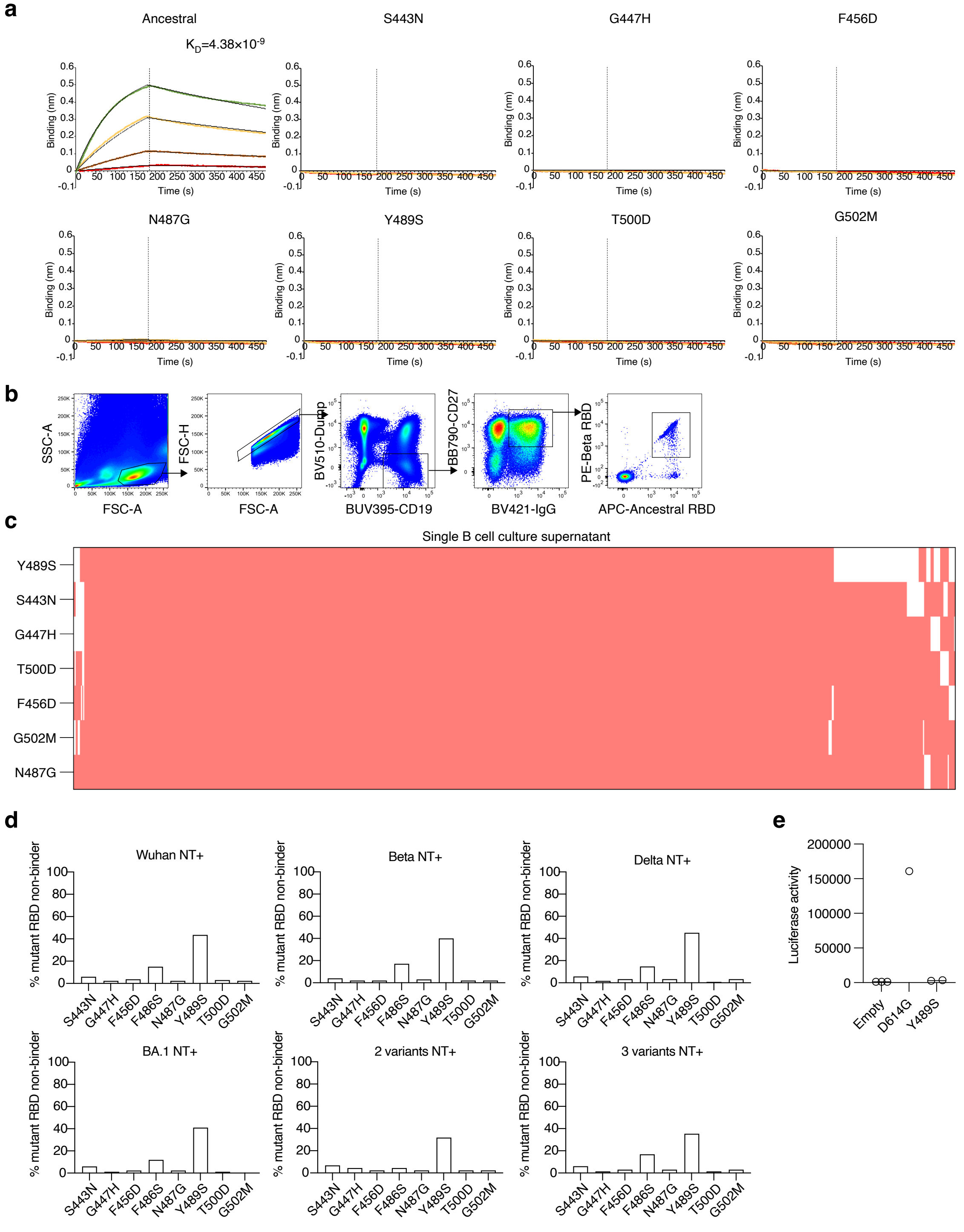
a
Ancestral
K_D=4.38×10^{-9}
S443N
G447H
F456D
N487G
Y489S
T500D
G502M
Binding (nm)
Time (s)
b
SSC-A
FSC-A
FSC-H
BV510-Dump
BUV395-CD19
BB790-CD27
BV421-IgG
PE-Beta RBD
APC-Ancestral RBD
c
Single B cell culture supernatant
Y489S
S443N
G447H
T500D
F456D
G502M
N487G
d
Wuhan NT+
Beta NT+
Delta NT+
BA.1 NT+
2 variants NT+
3 variants NT+
% mutant RBD non-binder
S443N
G447H
F456D
F486S
N487G
Y489S
T500D
G502M
e
Luciferase activity
Empty
D614G
Y489S

Supplementary Figure 1. Single B cell culture supernatant screening. a Binding affinity of hACE2 and recombinant RBDs were analyzed by bio-layer interferometry. **b** Sorting strategy for antigen-specific memory B cell sorting. Signals of CD2, CD4, CD10, CD14, IgD, and live/dead were detected in the "Dump" channel. **c** Binding of culture supernatants to each mutant RBDs is shown. Each line represents data from a single well. Wells with signals above the threshold are shown in red, and below the threshold are in white. **d** Percentages of culture supernatants with no binding to indicate mutant RBDs are shown. **e** Luciferase activity of pseudovirus containing indicated spike proteins is shown.

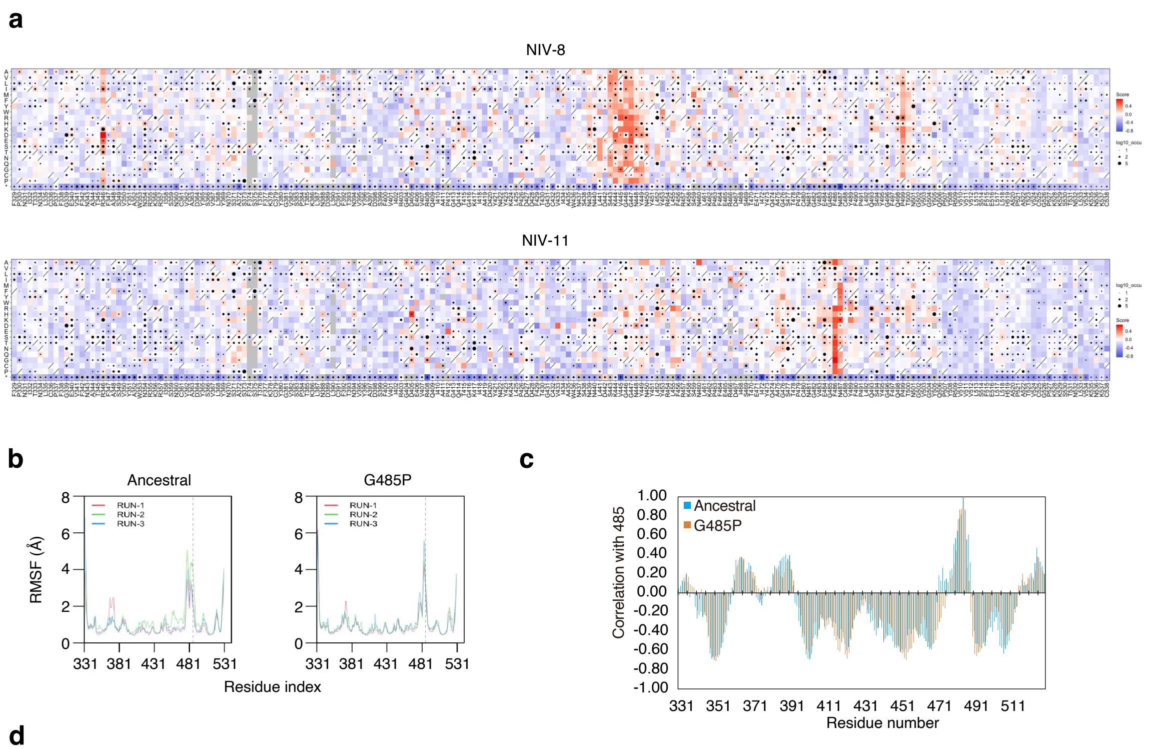

d

mAb ID	Mutation frequency (NGS)	Mutation (Sanger seq)
NIV-8	84.8%: K444M<br>14.5%: G447D	6/7 clones: K444M<br>1/7 clones: G447D
NIV-10	Not determined	No mutation
NIV-11	No mutation	No mutation
NIV-13	100%: F486S	8/8 clones: F486S

Supplementary Figure 2. Escape mutation analysis. a Full heat maps of escape fractions for NIV-8 and NIV-11 are shown. **b** Root mean square fluctuation of Cα atoms in the MD simulations. Dashed lines indicate the mutational site 485. **c** Cross correlations between residue at 485 and within the RBD in the MD simulations. **d** Escape variants were screened by passaging ancestral virus in the presence of the indicated monoclonal antibody. After limited-dilution cloning, RBD mutations were analyzed by next-generation sequencing (NGS) and by sanger sequencing.

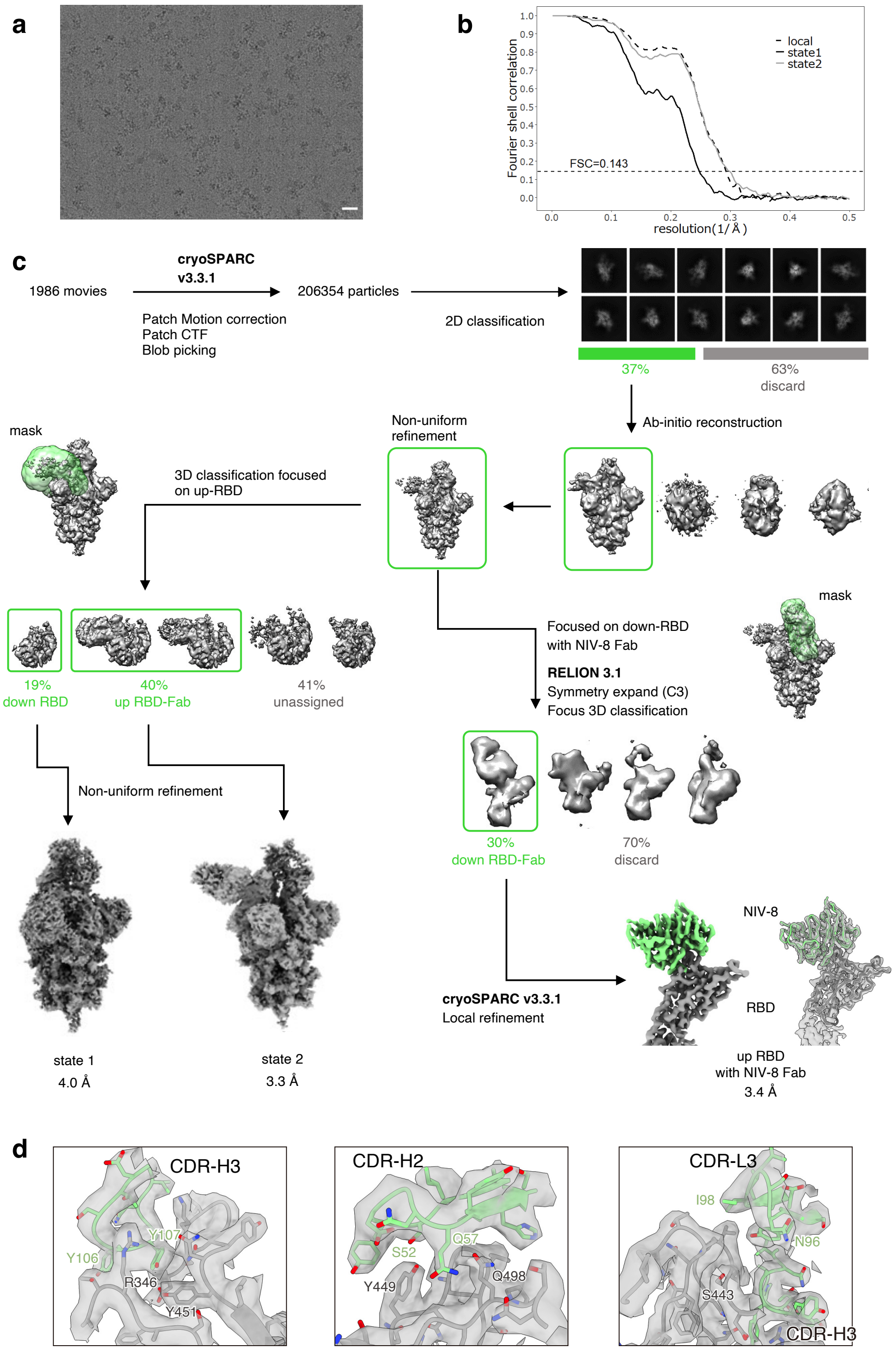

Supplementary Figure 3. Cryo-EM data processing of SARS-CoV-2 spike trimer complexed with NIV-8 Fab. a A representative raw cryo-EM micrograph (scale bar, 20 nm) from 1986 micrographs for this dataset. **b** Resolution assessment of the Spike-NIV-8 maps by Gold-standard

Fourier shell correlation (FSC) curves at 0.143 criteria. **c** Cryo-EM data processing flowchart. **d** The corresponding cryo-EM map fitting with the model around RBD and NIV-8 Fab interface.

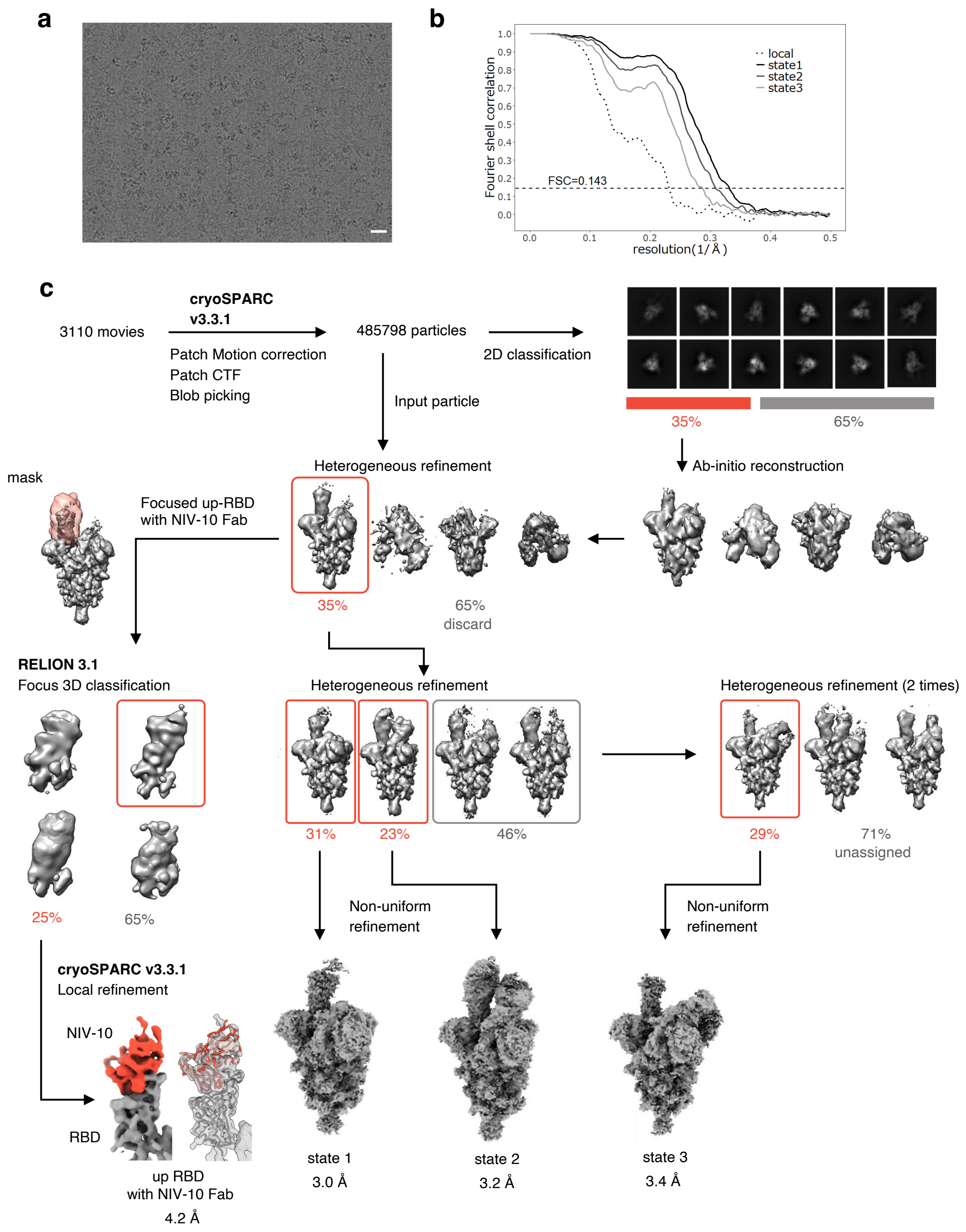

Supplementary Figure 4. Cryo-EM data processing of SARS-CoV-2 spike trimer complexed with NIV-10 Fab. a A representative raw cryo-EM micrograph (scale bar, 20 nm) from 3110 micrographs for this dataset. **b** Resolution assessment of the Spike-NIV-10 maps by Gold-standard Fourier shell correlation (FSC) curves at 0.143 criteria. **c** Cryo-EM data processing flowchart.

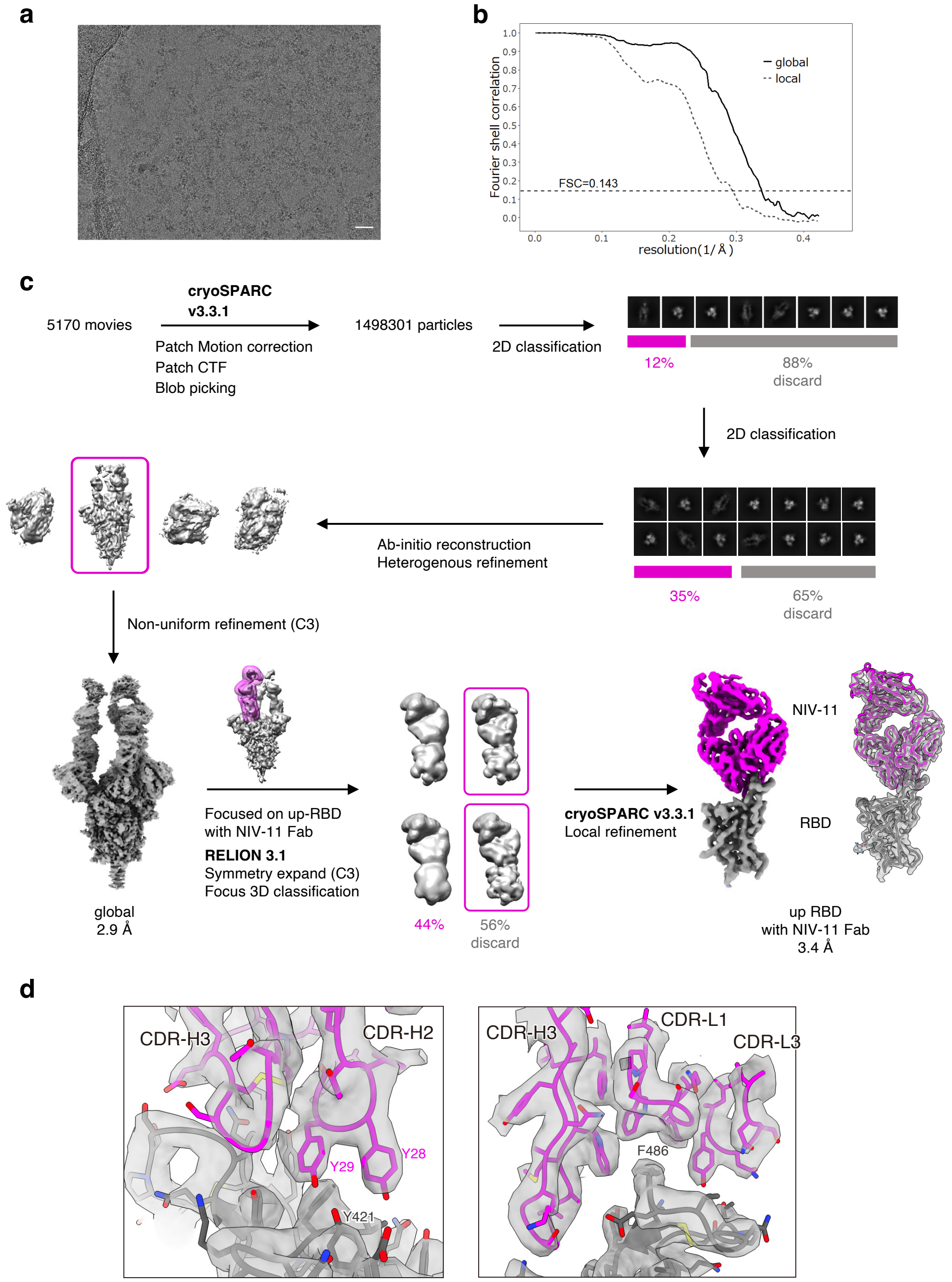

Supplementary Figure 5. Cryo-EM data processing of SARS-CoV-2 spike trimer complexed with NIV-11 Fab. a A representative raw cryo-EM micrograph (scale bar, 20 nm) from 5170 micrographs for this dataset. **b** Resolution assessment of the Spike-NIV-11 maps by Gold-standard Fourier shell correlation (FSC) curves at 0.143 criteria. **c** Cryo-EM data processing flowchart. **d** The corresponding cryo-EM maps fitting with the model around RBD and NIV-8 Fab interface.

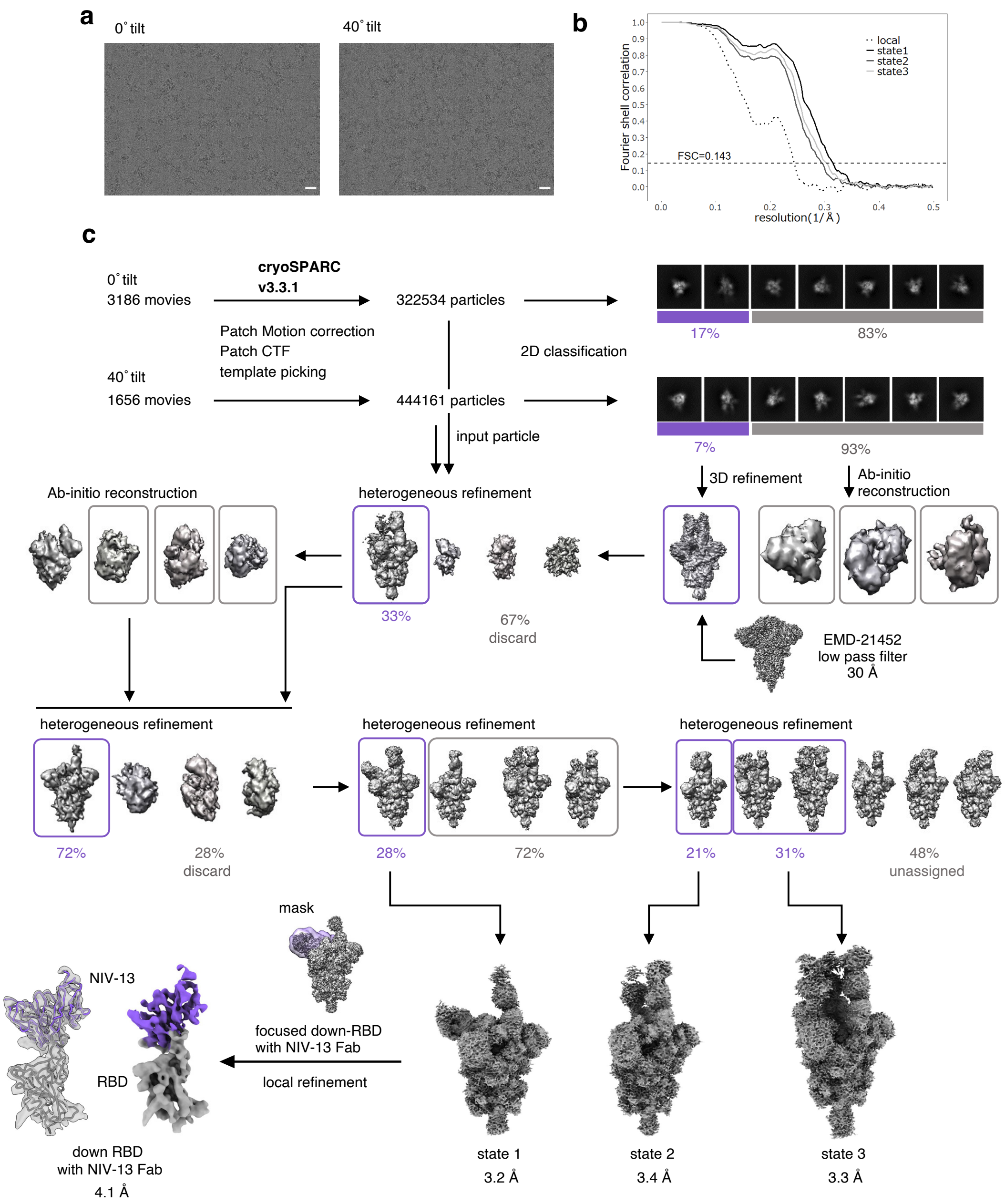

Supplementary Figure 6. Cryo-EM data processing of SARS-CoV-2 spike trimer complexed NIV-13 Fab. a Representative raw cryo-EM micrographs (scale bar, 20 nm) from 3186 and 1656 micrographs for the 0° (Left) and 40° tilt (Right) datasets, respectively. **b** Resolution assessment of the Spike-NIV-13 maps by Gold-standard Fourier shell correlation (FSC) curves at 0.143 criteria. **c** Cryo-EM data processing flowchart.

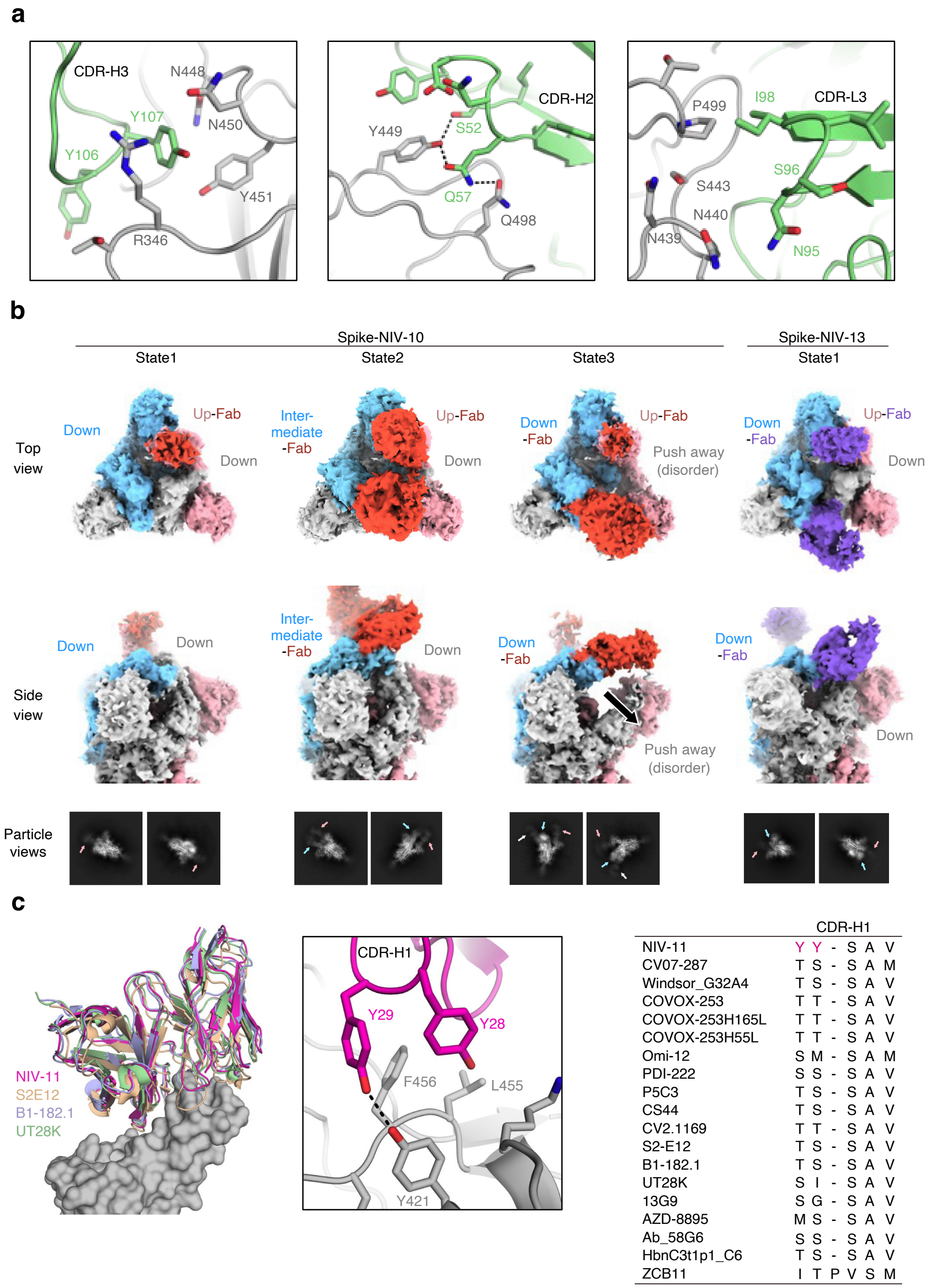

Supplementary Figure 7. Structural characterizations of spike protein in complex with Fabs. a Detailed recognition mode of CDR-H3 (left), CDR-H2 (middle), and CDR-L3 (right) of NIV-8. The dotted lines indicate polar interactions. **b** Top and side views of maps of each binding state of the Spike-NIV-10 and Spike-NIV-13 complexes. The particle views are also shown with arrows

indicating Fab bound to spike. The colors of the maps are indicated with the same color as in Fig. 5a. **c** (left) NIV-11 was superimposed on IGHV1-58/IGKV3-20 paired antibodies, S2E12, B1-182.1, UT28K. (middle) Detailed recognition mode of CDR-H1 of NIV-11. (right) Sequence alignment of CDR-H1 from IGHV1-58/IGKV3-20 paired antibodies.

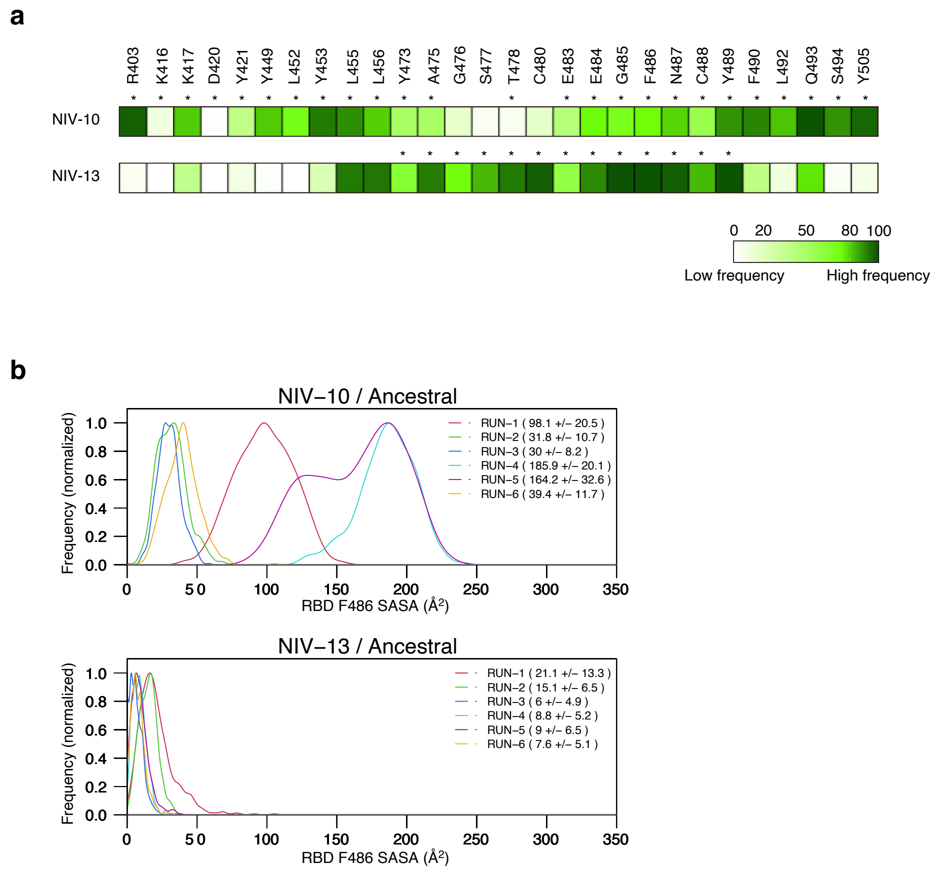

Supplementary Figure 8. MD simulations of RBD and Fabs.

a Frequency of contacts (4.5 Å cut-off) observed for the antibodies/RBD residue pairs in the MD simulations are shown as heatmaps. Asterisks represent epitope information observed in the crystal or cryo-EM structures. **b** Distributions of solvent accessible surface area (SASA) of RBD F486 in the MD simulations are shown. The SASA averages and the standard deviations of the last 50 ns trajectories are provided in parenthesis.

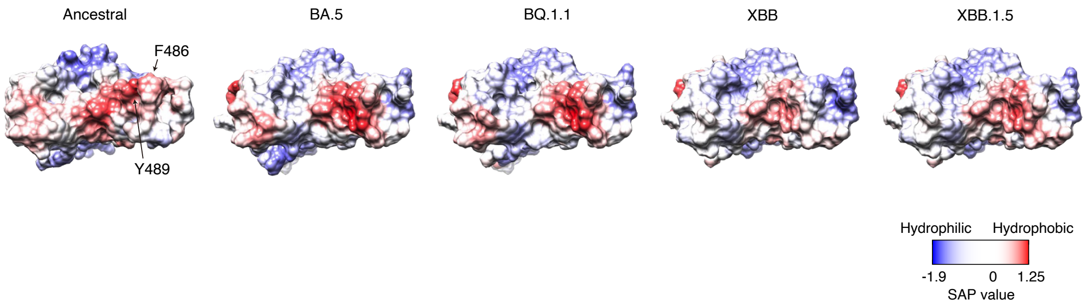

Supplementary Figure 9. SAP values for variant RBDs.

Surface representations of RBD variants are colored according to the SAP values. The viewing angle was the same as that shown in Fig. 6a.

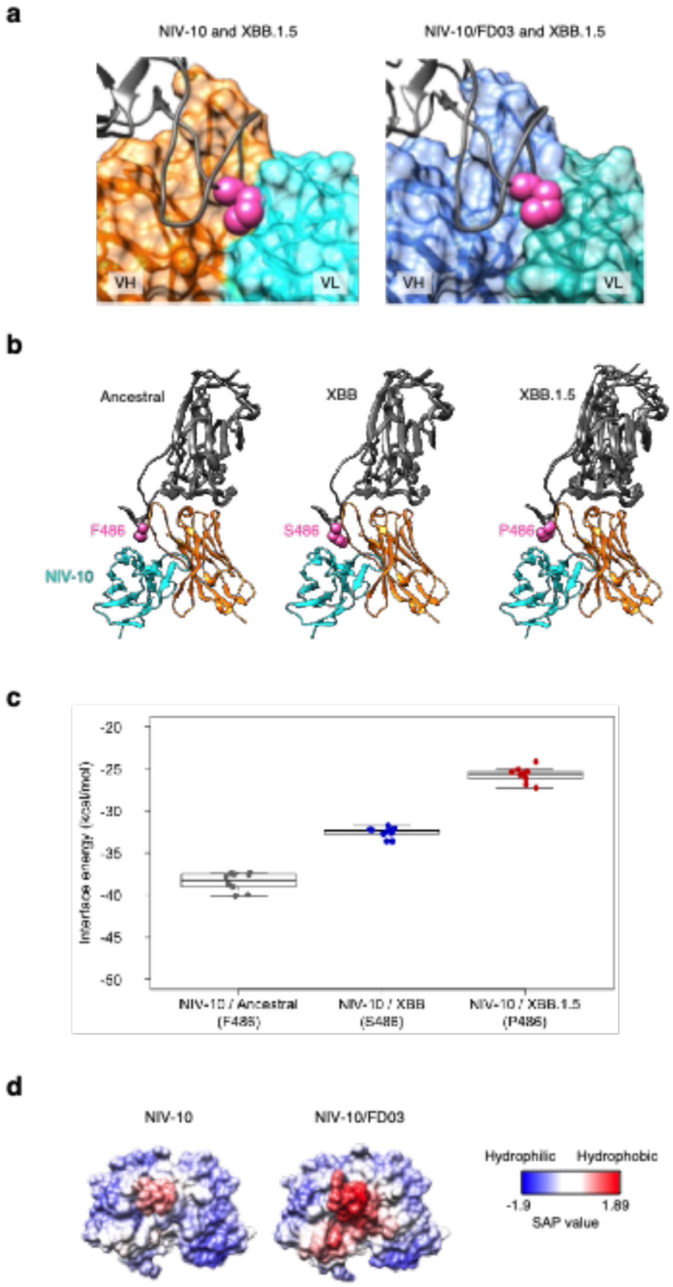

Supplementary Figure 10. Computational assessment of antibody-antigen interfaces.

a Structural comparison of XBB.1.5 with NIV-10 (left) and NIV-10/FD03 (right). P486 (pink ball) clashed with the light chain of NIV-10. **b** Overview of the predicted antibody-antigen complexes. XBB.1.5 exhibited greater structural variation. **c** Interface energies of the top 10 models (out of 20) determined by high-resolution docking simulations between NIV-10 and ancestral, XBB, or XBB.1.5

RBD. Each symbol represents one docking model. Box represents 1st quartile, median and 3rd quartile. The whiskers represent the minima and maxima. **d** Surface representations of the Fv region of NIV-10 and NIV-10/FD03 are colored according to the SAP values. The viewing angle is the same as that in Fig. 6b and c.

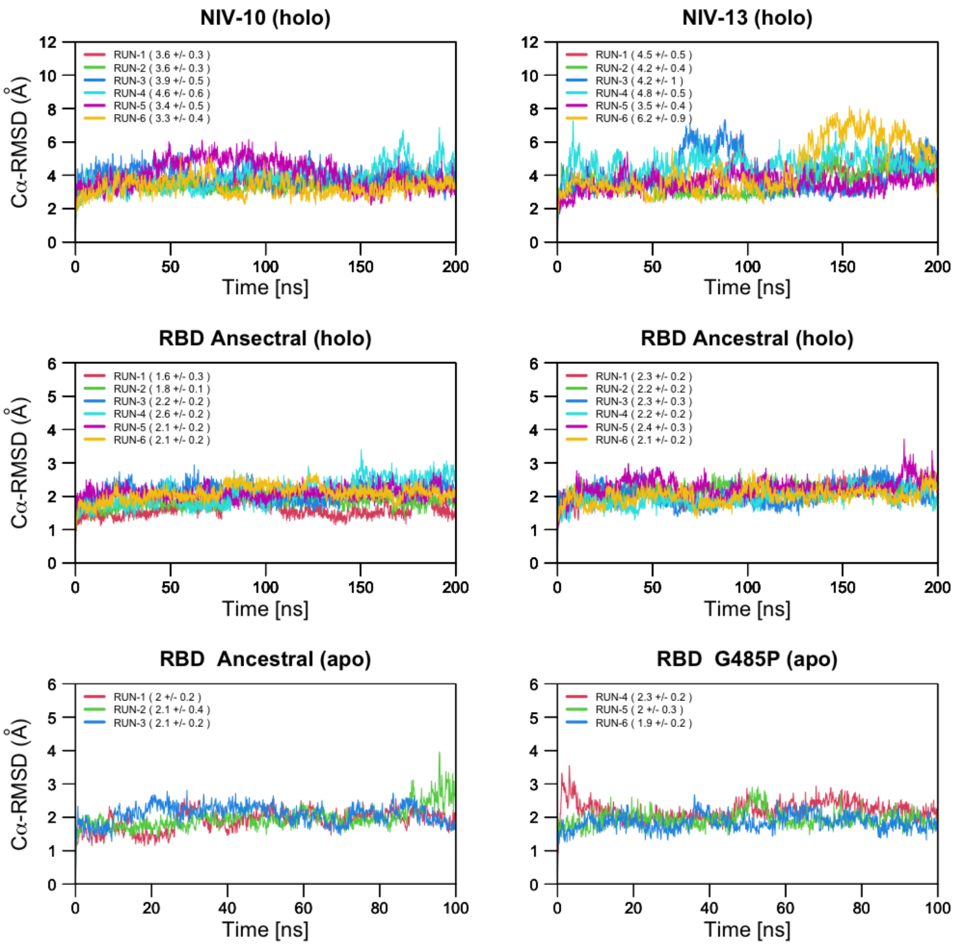

Supplementary Figure 11. Root mean square deviation (RMSD) of Cα atoms of the antibodies and RBD during each simulation. Averages and standard deviations of the last 50 ns of each simulation are shown in parenthesis.

Supplementary Table 1. Patient information

ID	Age	Sex	Time after infection (day)	Disease severity	Vaccine	Time after vaccination (day)
Tcc-028	49	Male	281	Mild	2x mRNA-1273	62
Tcc-029	38	Male	311	Moderate	-	-
Tcc-034	52	Female	223	Moderate	-	-

All volunteers provided written informed consent prior to enrolment.

Supplementary Table 2. Antibody V(D)J usage

mAb	HV	HJ	HD	H-CDR3 AA	LV	LJ	L-CDR3 AA	Patient ID
NIV-1	IGHV4-34	IGHJ4	IGHD5-12	ARGLYSGYDLAY	IGLV2-14	IGLJ1	SSYTSSNTYV	Tcc-029-d311 / Tcc-034-d223
NIV-5	IGHV5-51	IGHJ4	IGHD2-15	TRGGILCNGASCAADF	IGKV3-15	IGKJ5	QQYNNWPYT	Tcc-029-d311 / Tcc-034-d223
NIV-6	IGHV4-39	IGHJ4	IGHD3-3	TRLGGRGFTPEFDY	IGLV10-54	IGLJ3	SAWDITLSAWV	Tcc-029-d311 / Tcc-034-d223
NIV-8	IGHV3-30	IGHJ4	IGHD3-22	ARDGPDTSGYYANIYFDF	IGLV1-40	IGLJ3	QSYDNSLILAV	Tcc-029-d311 / Tcc-034-d223
NIV-9	IGHV3-49	IGHJ4	IGHD3-10	TRALPYVSGDY	IGLV1-44	IGLJ2	ATWDDSLNGPA	Tcc-029-d311 / Tcc-034-d223
NIV-10	IGHV3-7	IGHJ4	IGHD3-9	ARDGGYNILTAYYHAPSY	IGLV2-14	IGLJ2	SSYTGRSPYVV	Tcc-029-d311 / Tcc-034-d223
NIV-11	IGHV1-58	IGHJ3	IGHD2-15	AAPNCTGGSCYDGFNL	IGKV3-20	IGKJ1	QQYGSSPWL	Tcc-029-d311 / Tcc-034-d223
NIV-12	IGHV1-58	IGHJ3	IGHD2-15	AAPFCSGGSCYDGFNI	IGKV3-20	IGKJ1	QQYGSSPWT	Tcc-029-d311 / Tcc-034-d223
NIV-13	IGHV3-23	IGHJ1	IGHD3-22	AKGDYYDSSGYLAEYFPH	IGLV3-25	IGLJ2	QSTDISGVL	Tcc-029-d311 / Tcc-034-d223
NIV-15	IGHV2-5	IGHJ4	IGHD3-3	AQHTISKIFDH	IGLV2-14	IGLJ2	SSYTSGNLPLV	Tcc-029-d311 / Tcc-034-d223
NIV-20	IGHV3-53	IGHJ5	IGHD1-26	ARDLGVVGGTDQ	IGKV3-15	IGKJ2	QQYNNWPPGYT	Tcc-034-d223
NIV-22	IGHV3-9	IGHJ6	IGHD6-13	AKARFPGLAAAGYYGLDV	IGLV2-8	IGLJ2	SSYAGSNNLV	Tcc-034-d223
NIV-29	IGHV3-66	IGHJ5	IGHD1-26	ARDLGPVGATDQ	IGKV3-15	IGKJ2	QQYISWPPGYT	Tcc-028-d281
NIV-30	IGHV1-69	IGHJ6	IGHD1-1	ARVGGHWNDPFNYYGMDV	IGKV2-28	IGKJ1	MQALQTPT	Tcc-028-d281
NIV-35	IGHV1-58	IGHJ3	IGHD1-1	AAPHCNRTNCYDGFDL	IGKV3-20	IGKJ1	QQYGSSPWT	Tcc-028-d281
NIV-36	IGHV3-53	IGHJ4	IGHD1-26	ARDLGSVGGTDF	IGKV3-15	IGKJ2	QQYISWPPGYT	Tcc-028-d281

Supplementary Table 3. Antibody affinity.

		Ancestral	Delta	BA.1	BA.2
NIV-8	k_{on} (1/Ms)	1.06×10^{5}	2.69×10^{5}	1.26×10^{5}	1.03×10^{5}
	k_{off} (1/s)	1.15×10^{-6}	1.28×10^{-6}	3.12×10^{-3}	2.06×10^{-3}
	K_D (M)	1.09×10^{-11}	4.75×10^{-12}	2.47×10^{-8}	2.00×10^{-8}
NIV-10	k_{on} (1/Ms)	2.23×10^{5}	3.54×10^{5}	1.29×10^{5}	8.41×10^{4}
	k_{off} (1/s)	1.24×10^{-6}	1.31×10^{-6}	1.24×10^{-2}	6.40×10^{-3}
	K_D (M)	5.42×10^{-12}	3.71×10^{-12}	9.66×10^{-8}	7.61×10^{-8}
NIV-11	k_{on} (1/Ms)	5.36×10^{4}	1.06×10^{5}	3.66×10^{5}	3.22×10^{5}
	k_{off} (1/s)	1.12×10^{-6}	5.08×10^{-7}	3.35×10^{-3}	3.55×10^{-3}
	K_D (M)	2.08×10^{-11}	4.78×10^{-12}	9.14×10^{-9}	1.10×10^{-8}
NIV-13	k_{on} (1/Ms)	1.63×10^{5}	4.05×10^{5}	3.09×10^{5}	2.47×10^{5}
	k_{off} (1/s)	1.19×10^{-6}	3.72×10^{-5}	2.48×10^{-4}	3.69×10^{-4}
	K_D (M)	7.33×10^{-12}	9.19×10^{-11}	8.02×10^{-10}	1.49×10^{-9}

K_D of indicated Fabs and RBDs were determined by bio-layer interferometry.

Supplementary Table 4. List of amino acid substitutions that occur by single nucleotide mutations.

Position	Nucleotide code	Single nucleotide mutation (amino acid substitution)
T345	ACC	TCC (S), CCC (P), GCC (A), ATC (I), AAC (N), AGC (S)
R346	AGA	GGA (G), ATA (I), ACA (T), AAA (K), AGT (S), AGC (S)
R403	AGA	GGA (G), ATA (I), ACA (T), AAA (K), AGT (S), AGC (S)
D405	GAT	GTT (V), GCT (A), GGT (G), TAT (Y), CAT (H), AAT (N), GAA (E), GAG (E)
G416	GGA	GTA (V), GCA (A), GAA (E), CGA (R), AGA (R)
K417	AAG	ATG (M), ACG (T), AGG (R), CAG (Q), GAG (E), AAT (N), AAC (N)
D420	GAT	TAT (Y), CAT (H), AAT (N), GTT (V), GCT (A), GGT (G), GAA (E), GAG (E)
Y421	TAT	TTT (F), TCT (S), TGT (C), CAT (H), AAT (N), GAT (D)
N440	AAT	CAT (H), TAT (Y), GAT (D), ATT (I), ACT (T), AGT (S), AAA (K), AAG (K)
L441	CTT	TTT (F), ATT (I), GTT (V), CCT (P), CAT (H), CGT (R)
D442	GAT	TAT (Y), CAT (H), AAT (N), GTT (V), GCT (A), GGT (G), GAA (E), GAG (E)
S443	TCT	CCT (P), ACT (T), GCT (A), TTT (F), TAT (Y), TGT (C)
K444	AAG	ATG (M), ACG (T), AGG (R), CAG (Q), GAG (E), AAT (N), AAC (N)
V445	GTT	GCT (A), GAT (D), GGT (G), TTT (F), CTT (L), ATT (I)
G446	GGT	TGT (C), CGT (R), AGT (S)
G447	GGT	TGT (C), CGT (R), AGT (S)
N448	AAT	CAT (H), TAT (Y), GAT (D), ATT (I), ACT (T), AGT (S), AAA (K), AAG (K)
Y449	TAT	TTT (F), TCT (S), TGT (C), CAT (H), AAT (N), GAT (D)
N450	AAT	CAT (H), TAT (Y), GAT (D), ATT (I), ACT (T), AGT (S), AAA (K), AAG (K)
Y451	TAC	GGC (F), TCC (S), TGC (C), CAC (H), AAC (N), GAC (D)
L452	CTG	GTA (V), GCA (A), GAA (E), CGA (R), AGA (R)
Y453	TAT	TTT (F), TCT (S), TGT (C), CAT (H), AAT (N), GAT (D)
L455	TTG	TCG (S), TGG (W), ATG (M), GTG (V), TTT (F), TTC (F)
F456	TTT	TCT (S), TAT (Y), TGT (C), CTT (L), ATT (I), GTT (V), TTA (L), TTG (L)
Y473	TAT	TTT (F), TCT (S), TGT (C), CAT (H), AAT (N), GAT (D)
A475	GCC	GTC (V), GAC (D), GGC (G), TCC (S), CCC (P), ACC (T)

G476	GGT	TGT (C), CGT (R), AGT (S)
S477	AGC	ATC (I), ACC (T), AAC (N), TGC (C), CGC (R), GGC (G), AGA (R), AGG (R)
T478	ACA	ATA (I), AAA (K), AGA (R), TCA (S), CCA (P), GCA (A)
C480	TGT	TTT (F), TCT (S), TAT (Y), CGT (R), AGT (S), GGT (G), TGG (W)
V483	GTT	GCT (A), GAT (D), GGT (G), TTT (F), CTT (L), ATT (I)
E484	GAA	GTA (V), GCA (A), GGA (G), CAA (Q), AAA (K), GAT (D), GAC (D)
G485	GGT	TGT (C), CGT (R), AGT (S)
F486	TTT	TCT (S), TAT (Y), TGT (C), CTT (L), ATT (I), GTT (V), TTA (L), TTG (L)
N487	AAT	CAT (H), TAT (Y), GAT (D), ATT (I), ACT (T), AGT (S), AAA (K), AAG (K)
C488	TGT	TTT (F), TCT (S), TAT (Y), CGT (R), AGT (S), GGT (G), TGG (W)
Y489	TAC	GGC (F), TCC (S), TGC (C), CAC (H), AAC (N), GAC (D)
F490	TTT	TCT (S), TAT (Y), TGT (C), CTT (L), ATT (I), GTT (V), TTA (L), TTG (L)
L492	TTA	TCA (S), ATA (I), GTA (V), TTT (F), TTC (F)
Q493	CAA	CTA (L), CCA (P), CGA (R), AAA (K), GAA (E), CAT (H), CAC (H)
S494	TCA	TTA (L), CCA (P), ACA (T), GCA (A)
G496	GGT	TGT (C), CGT (R), AGT (S)
P499	CCC	CTC (L), CAC (H), CGC (R), TCC (S), ACC (T), GCC (A)
T500	ACT	ATT (I), AAT (N), AGT (S), TCT (S), CCT (P), GCT (A)
Y505	TAC	GGC (F), TCC (S), TGC (C), CAC (H), AAC (N), GAC (D)

Supplementary Table 5. Statistics of the Cryo-EM dataset and data processing

	Spike-NIV-8			Spike-NIV-10				Spike-NIV-11		Spike-NIV-13			
Structures	state1	state2	RBD-Fab	state1	state2	state3	RBD-Fab	global	RBD-Fab	state1	state2	state3	RBD-Fab
EMDB ID	EMD-33821	EMD-33822	EMD-33820	EMD-33824	EMD-33825	EMD-33826	EMD-33823	EMD-34741	EMD-34742	EMD-33828	EMD-33829	EMD-33830	EMD-33827
PDB ID	-	7YH7	7YH6	-	-	-	-	8HGL	8HGM	-	-	-	-
**Data collection**													
Microscope	Krios G4			Krios G4				Krios G4		Krios G4			
Detector	Gatan Biocontinuum energy filter with Gatan K3 direct electron detector			Gatan Biocontinuum energy filter with Gatan K3 direct electron detector				Gatan Biocontinuum energy filter with Gatan K3 direct electron detector		Gatan Biocontinuum energy filter with Gatan K3 direct electron detector			
Voltage (kV)	300			300				300		300			
Nominal magnification	130,000			130,000				130,000		130,000			
Defocusrange (µm)	-1.0 to -2.0			-1.0 to -2.0				-0.8 to -2.3		-1.0 to -2.0			
Physical pixel (Å)	0.67			0.67				0.67		0.67			
Electron dose (e/Å)	57.32			57.32				51.41		57.32			
Number of raw frames	50			50				50		50			
**Data processing**													
Extracted particles (n)	206354			485798				1498301		766695			
Particles for final map (n)	13430	27741	64245	51840	40003	21536	42213	61135	80132	84408	47157	69019	84408
Symmetry imposed	C1	C1	C1	C1	C1	C1	C1	C3	C1	C1	C1	C1	C1
Resolution	4.0	3.3	3.4	3.0	3.2	3.4	4.2	2.9	3.4	3.2	3.4	3.3	4.1
FSC threshold	0.143	0.143	0.143	0.143	0.143	0.143	0.143	0.143	0.143	0.143	0.143	0.143	0.143

Supplementary Table 6. Statistics of the Cryo-EM structure refinement and validation

	Spike-NIV-8 state 2 PDB 7YH7	Spike-NIV-8 RBD-Fab PDB 7YH6	Spike-NIV-11 global PDB 8HGL	Spike-NIV-11 RBD-Fab PDB 8HGM
Initial model used	PDB : 6VYB	PDB : 6M0J and predicted structure model using AlphaFold	PDB: 7K4N	PDB : 7K45 and predicted structure model using AlphaFold
**Model composition**				
Non-hydrogen atoms	28346	3343	34170	4886
Protein residues	3574	430	4317	632
Ligands	45	1	60	2
**R.m.s. deviations**				
Bond length (Å)	0.003	0.005	0.006	0.003
Bond angles (°)	0.502	0.629	0.640	0.579
**Validation**				
MolProbity score	1.56	1.89	1.76	1.68
Clash score	5.11	7.83	7.28	6.98
Rotamer outliers (%)	0	0	0	0
**Ramachandran plot**				
Outliers (%)	0	0.24	0	0
Allowed	4.21	7.08	5.29	4.31
Favored	95.79	92.69	94.71	95.69
**Model vs. Data**				
CC (mask)	0.83	0.85	0.80	0.85

Supplementary Table 7. Statistics of the crystal structure refinement and validation

	RBD_NIV-10 (PDB: 8HES)
Data collection	
Wavelength	1.0000
Resolution range	47.31 - 2.20 (2.27 - 2.20)
Space group	*P*212121
Unit cell	53.147 101.113 134.265 90 90 90
Total reflections	254148 (22170)
Unique reflections	36935 (3145)
Multiplicity	6.9 (7.0)
Completeness (%)	98.5 (98.9)
Mean I/sigma(I)	7.8 (1.5)
Wilson B-factor ($Å^2$)	38.47
R-merge	0.152 (1.588)
R-meas	0.164 (1.714)
R-pim	0.062 (0.639)
CC1/2	0.991 (0.722)
Refinement	
Reflections used in refinement	36857 (3629)
Reflections used for R-free	1785 (177)
R-work	0.1952 (0.2743)
R-free	0.2381 (0.3219)
Number of non-hydrogen atoms	4985
macromolecules	4735
ligands	14
solvent	236
Protein residues	620
RMS(bonds)	0.008
RMS(angles)	1.03
Ramachandran favored (%)	96.86
Ramachandran allowed (%)	2.97

Ramachandran outliers (%)	0.17
Rotamer outliers (%)	0.96
Clashscore	5.8
Average B-factor (Å^2)	44.17
macromolecules	44.2
ligands	62.59
solvent	42.65
Number of TLS groups	13
