# Supplementary material for: Structural delineation and computational design of SARS-CoV-2-neutralizing antibodies against Omicron subvariants
Source: Nat Commun. 2023 Jul 14;14:4198. doi: 10.1038/s41467-023-39890-8 (PMC10349087; doi:10.1038/s41467-023-39890-8)
Supplement: Supplementary file 3 — Reporting Summary [file 41467_2023_39890_MOESM3_ESM.pdf]

nature portfolio

Corresponding author(s): Saya Moriyama, Yoshimasa Takahashi

Last updated by author(s): Jun 16, 2023

Reporting Summary

Nature Portfolio wishes to improve the reproducibility of the work that we publish. This form provides structure for consistency and transparency in reporting. For further information on Nature Portfolio policies, see our Editorial Policies and the Editorial Policy Checklist.

Statistics

For all statistical analyses, confirm that the following items are present in the figure legend, table legend, main text, or Methods section.

n/a	Confirmed	
☐	☒	The exact sample size (*n*) for each experimental group/condition, given as a discrete number and unit of measurement
☐	☒	A statement on whether measurements were taken from distinct samples or whether the same sample was measured repeatedly
☐	☒	The statistical test(s) used AND whether they are one- or two-sided<br>*Only common tests should be described solely by name; describe more complex techniques in the Methods section.*
☒	☐	A description of all covariates tested
☒	☐	A description of any assumptions or corrections, such as tests of normality and adjustment for multiple comparisons
☐	☒	A full description of the statistical parameters including central tendency (e.g. means) or other basic estimates (e.g. regression coefficient) AND variation (e.g. standard deviation) or associated estimates of uncertainty (e.g. confidence intervals)
☒	☐	For null hypothesis testing, the test statistic (e.g. *F*, *t*, *r*) with confidence intervals, effect sizes, degrees of freedom and *P* value noted<br>*Give P values as exact values whenever suitable.*
☒	☐	For Bayesian analysis, information on the choice of priors and Markov chain Monte Carlo settings
☒	☐	For hierarchical and complex designs, identification of the appropriate level for tests and full reporting of outcomes
☒	☐	Estimates of effect sizes (e.g. Cohen's *d*, Pearson's *r*), indicating how they were calculated

Our web collection on statistics for biologists contains articles on many of the points above.

Software and code

Policy information about availability of computer code

Data collection	Molecular dynamics simulations of the RBD in complex with each antibody (NIV-10 and -13), as well as the RBD alone, were performed using GROMACS 2018.6 with the CHARMM36m force field. The initial structures of the NIV-10 and -13 complexes were obtained from the cryo-EM structures. Each system was solvated with TIP3P water in a rectangular box, so the minimum distance to the edge of the box was 10 Å under periodic boundary conditions. Na and Cl ions were added to neutralize the protein charge; next, additional ions were added to mimic a salt solution concentration of 0.15 M. Each system was energy-minimized for 5000 steps using steepest descent, heated from 50 to 310 K for 200 ps, and further equilibrations were continued for 500 ps with the NVT ensemble. During equilibration, positional restraint potentials were applied, and their force constants were gradually reduced. Further production runs were performed using the NPT ensemble. A cutoff distance of 12 Å was used for Coulomb and van der Waals interactions. The long-range electrostatics were evaluated using the particle mesh Ewald method. The LINCS algorithm was employed to constrain hydrogen atoms bonds. The time step was set as 2 fs throughout the simulation. A simulation was repeated six and three times for the antibody-RBD complex (200 ns each) and RBD (100 ns each) systems, respectively, which resulted in approximately 3 µs of aggregate simulation data, and the snapshots were saved every 100 ps. During the last 50 ns trajectories, the standard deviations of the root mean square deviation (RMSD) for the C atoms of the antibodies and RBD were within 1.0 Å. Therefore, to allow relaxation from the starting structures, all trajectory analyses were performed based on the last 50 ns trajectories, through the Gromacs, Prody, and MDTraj packages. Hydrophobicity was computed with the CHARMM free version 41b2. Docking and design calculations were performed with Rosetta 3.13.

Data analysis	CryoSPARC v3.3.1 was used for Cryo-EM image processing. Chimera (version 1.15), Chimera X (version 1.1), and PyMOL (version 2.3.3) were used for preparing figures. AlphaFold, Coot (version 0.9.6), Phenix (version 1.20) were used for cryo-EM model building/analysis and x-ray crystal structure analysis. IC50 calculation and statistical analysis were performed using Prism 9 (version 9.5.0). Immunoglobulin sequence data were analyzed using IgBlast (https://www.ncbi.nlm.nih.gov/igblast/).

For manuscripts utilizing custom algorithms or software that are central to the research but not yet described in published literature, software must be made available to editors and reviewers. We strongly encourage code deposition in a community repository (e.g. GitHub). See the Nature Portfolio guidelines for submitting code & software for further information.

Data

Policy information about availability of data

All manuscripts must include a data availability statement. This statement should provide the following information, where applicable:

- Accession codes, unique identifiers, or web links for publicly available datasets
- A description of any restrictions on data availability
- For clinical datasets or third party data, please ensure that the statement adheres to our policy

The DMS data generated in this study have been deposited at SpikeDB (https://sysimm.ifrec.osaka-u.ac.jp/sarscov2_dms/) and the National Center for Biotechnology Information Sequence Read Archive under BioProject ID PRJNA970973. Atomic coordinates and cryo-EM maps of the reported structure have been deposited into the Protein Data Bank and Electron Microscopy Data Bank (Supplementary Table 6). For SARS-CoV-2 spike in complex with NIV-8, states 1, 2, and RBD-Fab (local refinement) were assigned as EMD-33821 [https://www.ebi.ac.uk/emdb/EMD-33821], EMD-33822 [https://www.ebi.ac.uk/emdb/EMD-33822] (PDB 7YH7) [https://www.rcsb.org/structure/unreleased/7YH7], EMD-33820 [https://www.ebi.ac.uk/emdb/EMD-33820] (PDB 7YH6) [https://www.rcsb.org/structure/unreleased/7YH6], respectively. For SARS-CoV-2 spike in complex with NIV-10, states 1, 2, 3 and RBD-Fab (local refinement) were assigned as EMD-33824 [https://www.ebi.ac.uk/emdb/EMD-33824], EMD-33825 [https://www.ebi.ac.uk/emdb/EMD-33825], EMD-33826 [https://www.ebi.ac.uk/emdb/EMD-33826], EMD-33823 [https://www.ebi.ac.uk/emdb/EMD-33823], respectively. For SARS-CoV-2 spike in complex with NIV-10, the crystal structure of the RBD complex was assigned as PDB 8HES. For SARS-CoV-2 spike in complex with NIV-11, 3-up state and RBD-Fab (local refinement) were assigned as EMD-34741 [https://www.ebi.ac.uk/emdb/EMD-34741] (PDB 8HGL) [https://www.rcsb.org/structure/unreleased/8HGL], EMD-34732 [https://www.ebi.ac.uk/emdb/EMD-34732] (PDB 8HGM) [https://www.rcsb.org/structure/unreleased/8HGM], respectively. For SARS-CoV-2 spike in complex with NIV-13, states 1, 2, 3 and RBD-Fab (local refinement) were assigned as EMD-33828 [https://www.ebi.ac.uk/emdb/EMD-33828], EMD-33829 [https://www.ebi.ac.uk/emdb/EMD-33829], EMD-33830 [https://www.ebi.ac.uk/emdb/EMD-33830], respectively. The MD trajectories and the model structure of the FD03/XBB.1.5 RBD complex, along with the sequence information, have been submitted to the Biological Structure Model Archive (BSM-Arc) under BSM-ID BSM000046 [https://bsma.pdbj.org/entry/46]. All other data are available in the main text or the supplementary materials.

Human research participants

Policy information about studies involving human research participants and Sex and Gender in Research.

Reporting on sex and gender	Memory B cells / antibodies in peripheral blood samples were analyzed in the study. Sex data were not used.
Population characteristics	All convalescent samples were seropositive for nucleocapsid antibodies using Elecsys Anti-SARS-CoV-2.
Recruitment	SARS-CoV-2-infected individuals were enrolled at the Tokyo Center Clinic. Vaccinated individuals were enrolled at the Tokyo Metropolitan Bokutoh Hospital. We do not see bias that possibly could have negatively impacted on the results.
Ethics oversight	All studies were approved by the institutional review board of the National Institute of Infectious Diseases (#1132, #1321). This study was conducted in accordance with the principles of the Declaration of Helsinki. All volunteers provided written informed consent prior to enrolment.

Note that full information on the approval of the study protocol must also be provided in the manuscript.

Field-specific reporting

Please select the one below that is the best fit for your research. If you are not sure, read the appropriate sections before making your selection.

☒ Life sciences ☐ Behavioural & social sciences ☐ Ecological, evolutionary & environmental sciences

For a reference copy of the document with all sections, see nature.com/documents/nr-reporting-summary-flat.pdf

Life sciences study design

All studies must disclose on these points even when the disclosure is negative.

Sample size	For antibody screening, no sample size calculation was performed and all double-binding clones (823) we obtained were analyzed. For other experiments, sample size was determined based on previous studies.
Data exclusions	Data were excluded only for failed experiments.
Replication	ELISA, neutralization, BLI, in vivo infection, virus proliferation experiments were performed not less than twice as reported in the Data file.

Randomization	No randomization methods were used.
Blinding	Blinding was not relevant as the study was not based on subjective observations.

Reporting for specific materials, systems and methods

We require information from authors about some types of materials, experimental systems and methods used in many studies. Here, indicate whether each material, system or method listed is relevant to your study. If you are not sure if a list item applies to your research, read the appropriate section before selecting a response.

Materials & experimental systems

n/a	Involved in the study
☐	☒ Antibodies
☐	☒ Eukaryotic cell lines
☒	☐ Palaeontology and archaeology
☐	☒ Animals and other organisms
☒	☐ Clinical data
☒	☐ Dual use research of concern

Methods

n/a	Involved in the study
☒	☐ ChIP-seq
☐	☒ Flow cytometry
☒	☐ MRI-based neuroimaging

Antibodies

Antibodies used	Following antibodies were used for FACS analysis; BUV395-labeled anti-CD19 (BD Biosciences, 740287, clone HIB19), BV510-labeled anti-CD2 (BioLegend, 300218, clone RPA-2.10), CD4 (BioLegend, 300546, clone RPA-T4), anti-CD10 (BioLegend, 312220, clone HI10a), anti-CD14 (BioLegend, 301842, clone M5E2), anti-IgD (BioLegend, 348220, clone IA6-2), BV421-labeled anti-IgG (BD Biosciences, 562581, clone G18-145), live/dead aqua (Thermo Fisher Scientific, L34957), BB790-labeled anti-CD27 (BD Biosceinces, 624296, clone O323). For DMS, anti-hemagglutinin (HA) Alexa Fluor 647 (clone TANA2, MBL) was used. For ELISA, goat anti-human IgG-horseradish peroxidase (HRP, Southern Biotech) was used. For electrochemiluminescence immunoassay, SULFO-TAG-conjugated anti-human IgG (Meso-Scale Discovery) was used. NIV series antibodies were expressed using Expi293F and purified with a protein G column.
Validation	All primary antibodies used for FACS analysis were purchased from BD Biosciences and BioLegend, and was validated by manufacturer as described on their websites. In-house antibodies were validated by ELISA and neutralization assay.

Eukaryotic cell lines

Policy information about cell lines and Sex and Gender in Research

Cell line source(s)	Expi293F cells and HEK293T were used for protein expression. LentiX-293T cells were used for pseudovirus assay. VeroE6/TMPRSS2 cells were used for neutralization assay.
Authentication	Expi293F and LentiX-293 were purchased from manufacturer. VeroE6/TMPRSS2 cells were from JCRB cell bank.
Mycoplasma contamination	Cell lines were not tested for mycoplasma contamination.
Commonly misidentified lines (See ICLAC register)	No commonly misidentified cell lines were used.

Animals and other research organisms

Policy information about studies involving animals; ARRIVE guidelines recommended for reporting animal research, and Sex and Gender in Research

Laboratory animals	Syrian hamsters (4–5 weeks old females purchased from Japan SLC) were used in the study.
Wild animals	The study did not include wild animals.
Reporting on sex	Female hamsters were used for in vivo evaluation.
Field-collected samples	The study did not involve samples collected from the field.
Ethics oversight	Infection experiments were performed at the NIID BSL3 SPF animal facility in accordance with the guidelines of the Institutional Animal Care and Use Committee of NIID.

Note that full information on the approval of the study protocol must also be provided in the manuscript.

Flow Cytometry

Plots

Confirm that:

- [x] The axis labels state the marker and fluorochrome used (e.g. CD4-FITC).
- [x] The axis scales are clearly visible. Include numbers along axes only for bottom left plot of group (a 'group' is an analysis of identical markers).
- [x] All plots are contour plots with outliers or pseudocolor plots.
- [ ] A numerical value for number of cells or percentage (with statistics) is provided.

Methodology

Sample preparation	Blood samples were collected in Vacutainer CPT tubes (BD Biosciences), and PBMC and plasma samples were isolated via centrifugation according to the manufacturer's instruction.
Instrument	FACS Symphony S6 (BD Biosciences), MA900 cell sorter (Sony).
Software	Data were acquired by FACS Diva software and analyzed using FlowJo (version 10).
Cell population abundance	Single cell sorting was performed, and sorting purity was not determined.
Gating strategy	CD20+ Dump- IgG+ CD27+ Ancestral RBD binding+ Beta RBD binding+. The detailed gating scheme is showed in Supplementary figure 1c.

- [x] Tick this box to confirm that a figure exemplifying the gating strategy is provided in the Supplementary Information.
